# Supplementary material for: Dioscorea spp.: Comprehensive Review of Antioxidant Properties and Their Relation to Phytochemicals and Health Benefits
Source: Molecules. 2022 Apr 17;27(8):2530. doi: 10.3390/molecules27082530 (PMC9026138; doi:10.3390/molecules27082530)
Supplement: Supplementary file 1 [file molecules-27-02530-s001.zip › molecules-1601716-supplementary.pdf]

Abbreviations

AA: ascorbic acid
AAE: ascorbic acid equivalent
$ABTS^{•+}$: 2,2′ -azinobis-(3- ethylbenzothiazoline-6-sulfonate) scavenging capacity
AC: acetone
AcA: acetic acid
ACE: angiotensin converting enzyme
AIA: adjuvant-induced arthritis
AOM: azoxymethane
B: vitamin B
BDB: bark of D. batatas DECNE
BHT, butyl hydroxy toluene/ butylated hydroxytoluene
BuOH /*n*-BuOH: *n*-butanol
bw: body weight
C: vitamin C
Ca^{2+}, Mn^{2+}, Mg^{2+}: metal ions (M^{2+})
CAT: catalase
CE: catechin equivalents
CF: chloroform
CF–MeOH: chloroform-methanol
CGE: cyanidin glucoside equivalents
cGMP: cyclic guanosine 3′,5′-monophosphate
ChelA: chelating activity
COX-2: cyclooxygenase-2
CTC: carbon tetrachloride
CTX: cardiotoxin
CTX: cyclophosphamide
CUPRAC: cupric reducing antioxidant capacity
CWE, WWE, HWE: cold, warm and hot water extraction
Cy: cyanidin
CYCSE: Chinese yam cold-soaking extract
dw: dry weight
de: dried extract
DACNs: anthocyanins
DCM: dichloromethane
DE: diethyl ether
Diosgenin L, Diosgenin M, Diosgenin H: diosgenin dose: 20 mg/kg (low), 40 mg/kg (medium), 80 mg/kg (high)
DMBA: 7,12-dimethylbenz-[a]anthracene
DMSO: Dimethyl sulfoxide
DNA: deoxyribonucleic acid
$DPPH^{•}$: (2,2-diphenyl-1-picrylhydrazyl) scavenging capacity
E: extract
EA: ethyl acetate

EDTA: ethylene diamine tetra acetic acid
edw: extract dry weight
EF: EtOAc fraction
EPR: electron paramagnetic resonance
ESR: electron spin resonanc
EtOH: ethanol
EtOH-E: ethanol extract
eq: equivalent
Fe^{2+},Fe^{3+}: ferric ion
fr: fraction
FRAP: ferric reducing antioxidant power ability
FW: fresh weight
GAE: gallic acid equivalents
GA: gallic acid
GCL: glutamate-cysteine ligase
GC-MS: gas chromatography-mass spectroscopy
GI: glycemic index
GPx/ GSH-Px: glutathione peroxidase
GR: glutathione reductase
GSH: reduced glutathione; tGSH: total glutathione
GSSH: oxidized glutathione
GST: glutathione-S-transferase
HbAlc: glycated hemoglobin
HCl: hydrochloric acid
H_2O: water
H_2O_2: hydrogen peroxide
HCT: hydrocortisone
HFD: high fat diet
HPLC: High Performance Liquid Chromatography
HPLC-Q-TOF LC/MS: high-performance liquid chromatography coupled with quadrupole time-of-flight tandem mass spectrometry
HUVECs: human umbilical vein endothelial cells,
HX: hexane; *n*-hex: *n*-hexane
IC_{50}/EC_{50} effective concentration reducing 50% in the reaction present radicals
IBD: inflammatory bowel disease
IFN-γ : interferon-gamma
IL-1,IL-8,IL-1β, IL-6,: interleukin-1, interleukin-8, interleukin-1β, interleukin-6 (inflammation cytokines)
iNOS: inducible nitric oxide synthase
ISO: Isoprenaline/Isoproterenol
KDS-Yang: “kidney-yang deficiency syndrome”;
LCMS-IT-TOF: liquid chromatography mass connected with ion trap and time-of-flight-mass spectrometry;
LPI: lipid peroxidation inhibition
LPO: lipid peroxide level

LPS: lipopolysaccharide
MDA: malondialdehyde
MeOH: methanol
MeOH-E: methanol extract
MPYF: modified process of yam flour
TPYF: traditional process of yam flour
MW: molecular weight
NADES: natural depp eutectic solvent
NALP3: inflammasome
ND: not detected
NF-κB: nuclear factor-Kb
NF kappa B: nuclear factor kappa B
NO: nitric oxide
NO_2: nitrogen dioxide radical
$^{\bullet}NO$: nitric oxide scavenging activity
Nrf2: nuclear factor-erythroid 2-related factor 2
O_2^{-}: superoxide radical scavenging activity
$O_2^{\bullet -}$: superoxide anion scavenging activity
$^{\bullet}OH$: hydroxyl radical scavenging activity
ORAC: oxygen radical absorbance capacity
PBZ: paclobutrazol
pdw/dw: plant dry weight/dry weight
PE: petrol ether;
pfw: plant fresh weight
Pg: pelargonidin
PGE2: prostaglandin E2
Pn: Peonidin
$PTIO^{\bullet}$: phenyl-4,4,5,5-tetramethylimidazoline-1-oxyl 3-oxide
Q: quercetin
QE: quercetin equivalents
RE: rutin equivalents
RMD: red mold dioscorea
RDN: rhizoma Dioscoreae nipponicae
ROS: reactive oxygen species
RP: reducing power
RSA: radical scavenging activity
Ref: reference
SNP: sodium nitroprusside
SOD: superoxide dismutase,
STZ: streptozotocin
TAC: total anthocyanin content
T-AOC, total antioxidant capacity
TBA: thiobarbituric acid
TBARS: thiobarbituric acid-reacting substances

*t*BHP: *tert*-butyl hydroperoxide,
TE: Trolox (6-hydroxy-2,5,7,8-tetramethylchroman-2-carboxylic acid) equivalent
TFC: total flavonoid content
TFL: total flavonol content
TGF-β1: transforming growth factor-β1
TNBS: trinitrobenzenesulfonic acid
TNF-α: tumor necrosis factor-α (inflammation cytokines)
TPC: total phenol content
TPYF: prepared by traditional process
TS: total saponins
TSS: total steroid saponins
TT: total tanin
UAE: ultrasonic-assisted extraction
YP: yellow powder
UPLC-DAD-Q-TOF-MS/MS: ultra-performance liquid chromatography with diode array detection and quadrupole time-of-flight tandem mass spectrometry
α-Toc: α-tocopherol
2,7-dOH-4,6-dMetOP: 2,7-dihydroxy-4,6-dimethoxy phenanthrene
6,7-dOH -2,4-dMetOP: 6,7-dihydroxy-2,4-dimethoxy phenanthrene
6-OH-2,4,7-tMetOP: 6-hydroxy-2,4,7-trimethoxyphenanthrene
